# Stromal expression of ALDH1 in human breast carcinomas indicates reduced tumor progression

**DOI:** 10.18632/oncotarget.4628

**Published:** 2015-07-20

**Authors:** Natalia Bednarz-Knoll, Paulina Nastały, Anna Żaczek, Małgorzata Stoupiec, Sabine Riethdorf, Harriet Wikman, Volkmar Müller, Jarosław Skokowski, Jolanta Szade, Aleksandra Sejda, Marzena Wełnicka-Jaśkiewicz, Klaus Pantel

**Affiliations:** ^1^ Department of Tumor Biology, University Medical Center Hamburg-Eppendorf, Hamburg, Germany; ^2^ Laboratory of Cell Biology, Department of Medical Biotechnology, Intercollegiate Faculty of Biotechnology, University of Gdańsk and Medical University of Gdańsk, Gdańsk, Poland; ^3^ Department of Gynecology, University Medical Center Hamburg-Eppendorf, Hamburg, Germany; ^4^ Bank of Frozen Tissues & Genetic Specimens, Medical University of Gdańsk, Gdańsk, Poland; ^5^ Department of Surgical Oncology, Medical University of Gdańsk, Gdańsk, Poland; ^6^ Department of Pathomorphology, Medical University of Gdańsk, Gdańsk, Poland; ^7^ Department of Oncology and Radiotherapy, Medical University of Gdańsk, Gdańsk, Poland

**Keywords:** breast cancer, ALDH1, intra-tumoral stroma, tumor-stroma interactions, tumor progression

## Abstract

Interactions between cancer cells and microenvironment are emerging issue in tumor progression. Aldehyde dehydrogenase 1 (ALDH1) is a recognized cancer stem cell marker but little is known about its role in intratumoral stroma. Therefore, we focused on ALDH1 expression in tumor-associated stroma of breast carcinomas (BrCa).

Stromal and tumoral ALDH1 expression was evaluated immunohistochemically in BrCa and their lymph node metastases (LNMs), and related to clinico-pathological characteristics, patients’ outcome, presence of CD68, HLADR, retinoic acid (RA) in stroma, and selected proteins in tumor cells.

ALDH1(+) stromal cells were detected in 53% of 374 BrCa and 61% of 102 LNMs. ALDH1(+) stroma in primary tumor correlated to longer disease-free (*p* = 0.030), metastasis-free (*p* = 0.024), and overall survival (*p* = 0.043) having an independent prognostic impact on DFS (multivariate analysis, *p* = 0.047). It was associated with concomitant presence of HLA-DR(+) stromal cells and RA in tumor cells (both *p* < 0.001), and inversely associated with vimentin expression in tumor cells (*p* = 0.036). ALDH1(+) stroma in LNMs correlated inversely to presence of disseminated tumor cells in patients’ bone marrow (*p* = 0.014) and was independent prognosticator of shorter DFS and MFS (multivariate analysis, *p* = 0.004 and *p* = 0.002, respectively).

In conclusion, ALDH1 expression in tumor-associated stromal cells indicates reduced BrCa progression, possibly via RA secretion.

## INTRODUCTION

A current model of tumor development assumes the existence of cancer stem cells that might be the drivers of primary tumor growth and probably also the initiators of metastases, and appear to be resistant to applied therapies including radiotherapy and chemotherapy [1]. Cancer stem cells are identified by certain markers but the expression of these markers is not restricted to tumor cells. Moreover, the biology behind some of these markers is not fully understood yet.

The cytoplasmic protein aldehyde dehydrogenase (ALDH1), originally described as a detoxification enzyme [2], has been introduced as a putative ubiquitous marker of cancer stem cells both *in vitro* and *in situ* [1, 3–10]. Although most frequently investigated in breast cancer, ALDH1 has been also detected in colorectal [11, 12], lung [13], ovarian [14], bladder [5] and more recently in pancreatic [7, 15], prostate [8], and esophageal squamous cell carcinoma [16]. ALDH1 expression in tumor cells has been shown to be associated with unfavorable clinical outcome in these different types of tumors [3, 4, 8, 11–13, 15–19]. Of note, its expression has been found in circulating tumor cells of breast and colorectal cancer patients [20–22], particularly of those not responding to systemic therapy aimed to kill metastatic cells [20].

Little is known about the presence of ALDH1 in the microenvironment of solid tumors [14, 23–27]. The prevalence, origin and role of ALDH1(+) stromal cells in normal tissues and cancers remain largely unknown. ALDH1 is involved in the latter steps of the synthesis of retinoic acid, which, in turn, might e.g. inhibit proliferation and migratory abilities of tumor cells as well as induce their differentiation [28–30]. In normal human mammary epithelium ALDH1 was shown to affect proliferation and differentiation of stem/progenitor cells via its function in retinoic acid metabolism [31]. In guts retinoic acid derived from ALDH1(+) dendritic cells was observed to activate immune cells [32]. Thus, it is conceivable that if present in tumors ALDH1(+) stromal cells might synthesize and secrete retinoic acid leading to cancer cell differentiation and reduced tumor aggressiveness.

In the current study, we have focused on the clinical relevance of ALDH1 expression in breast cancer-associated stromal cells present in primary tumors and their regional lymph node metastases. Moreover, we undertook a first attempt to unravel the biology behind ALDH1 expression in intratumoral stroma cells.

## RESULTS

### ALDH1 expression in stromal cells of primary breast carcinomas and lymph node metastases

Three-hundred-seventy-four breast cancer patients and LNM samples from 102 patients were informative for ALDH1 staining both in tumor and stromal cells. Fifty-eight patients were informative for ALDH1 staining in both primary tumor and corresponding LNM (matched pairs).

**Figure 1:**
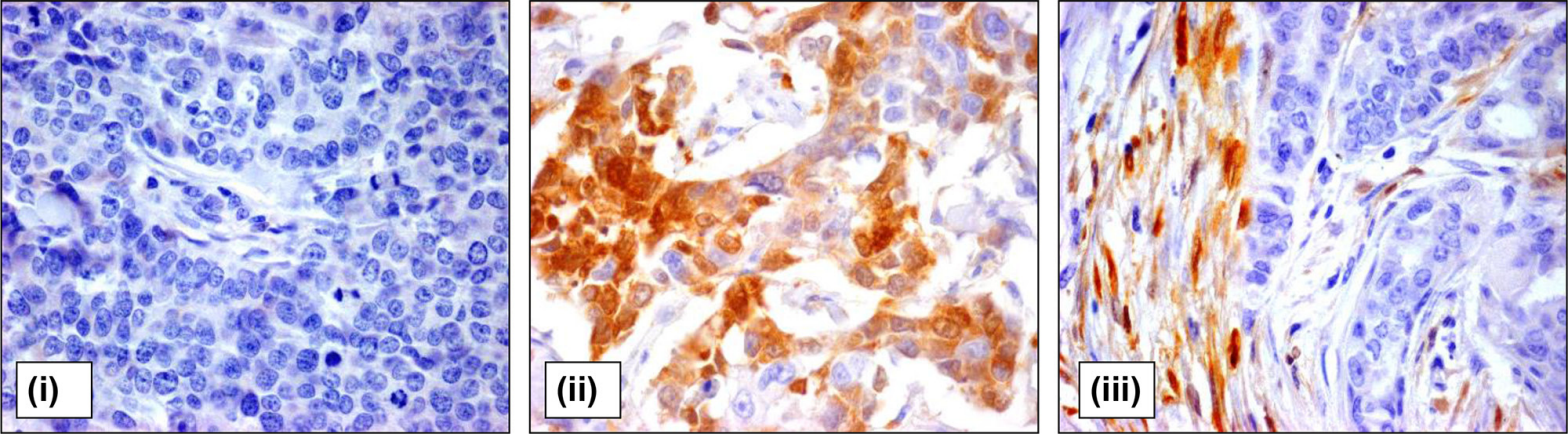
ALDH1 expression in tumor and stromal cells of breast cancer patients Representative pictures of breast cancer samples with tumor cells negative for ALDH1 staining **(i)**, high percentage of ALDH1-positive tumor cells **(ii)**, and ALDH1-positive stromal cells **(iii)**. Magnification 400x.

Intratumoral stromal ALDH1 expression was found in 197 (52.7%) and 62 (60.8%) breast cancer patients in primary tumors and LNMs, respectively. If present, ALDH1 was detected as moderate or strong cytoplasmic staining in spindle- and/or polygonal-like shaped stromal cells located between and/or around tumor cells (Figure 1).

The expression of ALDH1 in stromal cells of LNMs was significantly correlated to its expression in primary tumors (*n* = 58, R^2^ = 0.294, *p* = 0.025). Among 58 matched PT-LNM pairs, 36 (62.1%) displayed similar ALDH1 staining in stromal cells at both sites, whereas 17 (29.3%) patients had ALDH1-positive stromal cells exclusively in LNM and only 3 (8.6%) patients had ALDH1-positive stromal cells exclusively in the primary tumor.

### Associations of ALDH1 expression in stromal cells to clinico-pathological parameters and patients’ outcome

Expression of ALDH1 in stromal cells did not correlate to any clinico-pathological parameter (Suppl. Table 1) but had a significant impact on patients’ outcome. It correlated inversely to disease recurrence (Chi^2^ = 4.056, *p* = 0.044) and cancer-related death (Chi^2^ = 4.460, *p* = 0.035) (Suppl. Table 1).

**Figure 2:**
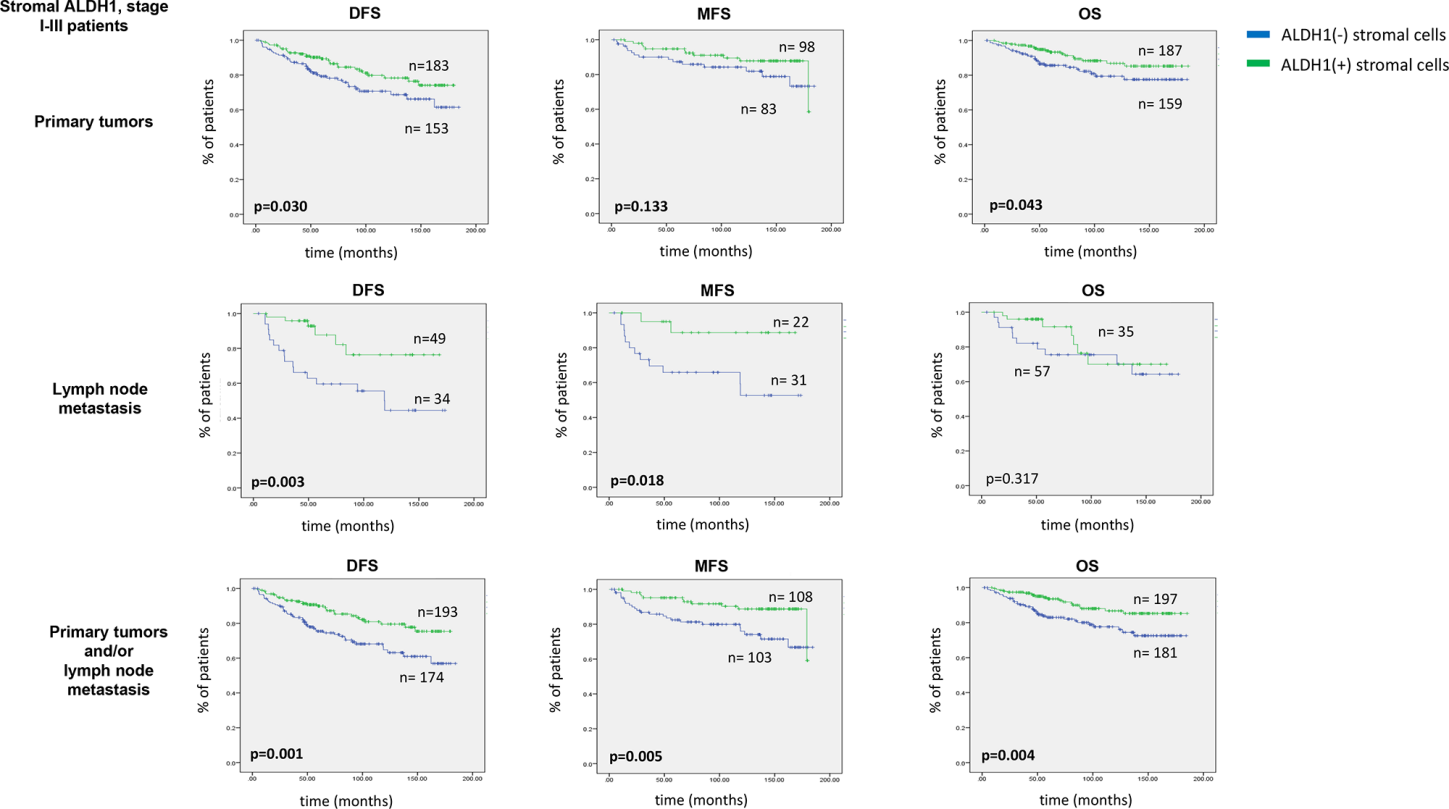
Impact of stromal ALDH1 expression on survival of stage I-III breast cancer patients DFS indicates disease free survival, MFS - metastasis free survival OS – overall survival. Of note, DFS and OS analysis was performed in all patients, whereas MFS analysis - in Hamburg cohort

Patients’ survival data were available for up to 15 years. Survival analyses were performed in stage I-III patients. Stromal ALDH1 staining evaluated in primary tumors indicated longer disease-free and overall survival (Kaplan-Meier log rank analysis, *p* = 0.030 and *p* = 0.043, respectively) (Figure 2). Stromal ALDH1 staining evaluated in lymph node metastasis indicated longer disease-free and metastasis-free survival (Kaplan-Meier log rank analysis, *p* = 0.003 and *p* = 0.018, respectively) (Figure 2). Stromal ALDH1 staining evaluated in primary tumors and/or lymph node metastasis indicated longer disease-free, metastasis-free and overall survival (Kaplan-Meier log rank analysis, *p* = 0.001, *p* = 0.005 and *p* = 0.004, respectively) (Figure 2).

In order to validate the prognostic value of ALDH1 staining in stroma, T and N status, grading score and/or hormone receptor status as well as ALDH1 expression were included in the multivariate analysis depending on the subanalyses (Table 1). Lymph node metastasis was the strongest independent predictor of disease-free and overall survival (Table 1).

**Table 1:** Multivariate analysis of ALDH1 and clinico-pathological markers in stage I-III breast cancer patients

A						
PT						
**DFS, *n* = 328**						
	Univariate analysis			Multivariate analysis		
	*p*-value	HR	95% CI for HR	*p*-value	HR	95% CI for HR
**T status (T3-4 vs. T1-2)**	**<0.001**	**1.683**	**1.297–2.183**	0.100	1.322	0.948–1.844
**N status (N1 vs. N0)**	**<0.001**	**2.892**	**1.950–4.288**	**0.001**	**2.413**	**1.453–4.007**
**Grading (G3 vs. G1-2)**	**0.009**	**1.295**	**1.067–1.572**	0.731	1.045	0.813–1.343
**Age (≥58 vs. <58)**	0.628	0.909	0.618–1.337	–	–	–
**Hormone receptor status (pos vs. neg)**	**0.007**	**0.559**	**0.366–0.856**	0.137	0.667	0.392-1.137
**Her-2 status (pos vs. neg)**	0.135	1.690	0.850–3.363	–	–	–
**stromal ALDH1 (neg vs. pos)**	**0.032**	**0.841**	**0.717–0.985**	**0.047**	**0.851**	**0.726–0.928**

PT						
OS, *n* = 330						
	Univariate analysis			Multivariate analysis		
	*p*-value	HR	95% CI for HR	*p*-value	HR	95% CI for HR
**T status (T3-4 vs. T1-2)**	**0.016**	**1.605**	**1.093–2.357**	0.248	1.264	0.850–1.879
**N status (N1 vs. N0)**	**<0.001**	**3.555**	**1.881–6.718**	**<0.001**	**3.247**	**1.682–6.266**
**Grading (G3 vs. G1-2)**	0.511	1.107	0.817–1.500	–	–	–
**Age (≥58 vs. <58)**	0.458	1.251	0.692–2.262	–	–	–
**Hormone receptor status (pos vs. neg)**	0.082	0.570	0.302–1.075	–	–	–
**Her-2 status (pos vs. neg)**	0.399	0.426	0.058–3.103	–	–	–
**stromal ALDH1 (neg vs. pos)**	**0.042**	**0.810**	**0.662–0.992**	0.051	0.817	0.667–1.001

B						
LNM						
DFS, *n* = 79						
	Univariate analysis			Multivariate analysis		
	*p*-value	HR	95% CI for HR	*p*-value	HR	95% CI for HR
**T status (T3-4 vs. T1-2)**	**<0.001**	**1.683**	**1.297–2.183**	0.167	1.569	0.828–2.972
**Grading (G3 vs. G1-2)**	**0.009**	**1.295**	**1.067–1.572**	0.073	1.506	0.962–2.356
**Age (≥58 vs. <58)**	0.628	0.909	0.618–1.337	–	–	–
**Hormone receptor status (pos vs. neg)**	**0.007**	**0.559**	**0.366–0.856**	**0.031**	**3.679**	**1.125–12.037**
**Her-2 status (pos vs. neg)**	0.135	1.690	0.850–3.363	−	−	−
**stromal ALDH1 (neg vs. pos)**	**0.005**	**0.639**	**0.466–0.876**	**0.004**	**0.627**	**0.454–0.865**

LNM						
MFS, *n* = 51						
	Univariate analysis			Multivariate analysis		
	*p*-value	HR	95% CI for HR	*p*-value	HR	95% CI for HR
**T status (T3-4 vs. T1-2)**	**0.009**	**1.710**	**1.143–2.560**	**0.042**	**2.087**	**1.026–4.244**
**Grading (G3 vs. G1-2)**	**0.048**	**1.340**	**1.003–1.791**	0.614	0.849	0.449–1.604
**Age (≥58 vs. <58)**	0.480	0.811	0.454–1.449	–	–	–
**Hormone receptor status (pos vs. neg)**	0.733	0.875	0.407–1.880	–	–	–
**Her-2 status (pos vs. neg)**	0.766	0.806	0.195–3.336	−	–	−
**tumoral ALDH1 (pos vs. neg)**	**0.016**	**4.244**	**1.314–13.705**	**<0.001**	**24.426**	**4.208–141.793**
**stromal ALDH1 (neg vs. pos)**	**0.034**	**0.583**	**0.354–0.960**	**0.002**	**0.405**	**0.226–0.725**

C						
PT+LNM						
DFS, *n* = 359						
	Univariate analysis			Multivariate analysis		
	*p*-value	HR	95% CI for HR	*p*-value	HR	95% CI for HR
**T status (T3-4 vs. T1-2)**	**<0.001**	**1.683**	**1.297–2.183**	0.068	1.316	0.980-1.767
**N status (N1 vs. N0)**	**<0.001**	**2.892**	**1.950–4.288**	**<0.001**	**2.476**	**1.527–4.013**
**Grading (G3 vs. G1-2)**	**0.009**	**1.295**	**1.067–1.572**	0.605	1.063	0.843-1.342
**Age (≥58 vs. <58)**	0.628	0.909	0.618–1.337	–	–	–
**Hormone receptor status (pos vs. neg)**	**0.007**	**0.559**	**0.366–0.856**	0.188	0.718	0.439–1.175
**Her-2 status (pos vs. neg)**	0.135	1.690	0.850–3.363	–	–	–
**stromal ALDH1 (neg vs. pos)**	**0.001**	**0.782**	**0.673–0.910**	**0.005**	**0.806**	**0.693–0.938**

PT+LNM						
MFS, *n* = 204						
	Univariate analysis			Multivariate analysis		
	*p*-value	HR	95% CI for HR	*p*-value	HR	95% CI for HR
**T status (T3-4 vs. T1-2)**	**0.009**	**1.710**	**1.143–2.560**	0.145	1.421	0.886–2.279
**N status (N1 vs. N0)**	**<0.001**	**3.269**	**1.815–5.887**	**0.023**	**2.358**	**2.123–4.949**
**Grading (G3 vs. G1-2)**	**0.048**	**1.340**	**1.003–1.791**	0.866	1.033	0.711–1.500
**Age (≥58 vs. <58)**	0.480	0.811	0.454–1.449	–	–	–
**Hormone receptor status (pos vs. neg)**	0.733	0.875	0.407–1.880	–	–	–
**Her-2 status (pos vs. neg)**	0.766	0.806	0.195–3.336	–	–	–
**tumoral ALDH1 (pos vs. neg)**	**0.016**	**2.778**	**1.212–6.366**	**0.015**	**3.310**	**1.264–8.663**
**stromal ALDH1 (neg vs. pos)**	**0.007**	**0.722**	**0.569–0.916**	**0.007**	**0.712**	**0.555–0.913**

PT+LNM						
OS, *n* = 332						
	Univariate analysis			Multivariate analysis		
	*p*-value	HR	95% CI for HR	*p*-value	HR	95% CI for HR
**T status (T3-4 vs. T1-2)**	**0.016**	**1.605**	**1.093–2.357**	0.265	1.254	0.843–1.865
**N status (N1 vs. N0)**	**<0.001**	**3.555**	**1.881–6.718**	**0.001**	**3.192**	**1.653–6.164**
**Grading (G3 vs. G1-2)**	0.511	1.107	0.817–1.500	–	–	–
**Age (≥58 vs. <58)**	0.458	1.251	0.692–2.262	–	–	–
**Hormone receptor status (pos vs. neg)**	0.082	0.570	0.302–1.075	–	–	–
**Her-2 status (pos vs. neg)**	0.399	0.426	0.058–3.103	–	–	–
**stromal ALDH1 (neg vs. pos)**	**0.03**	**0.797**	**0.649–0.978**	0.051	0.815	0.664–1.001

A - for ALDH1 evaluated in primary tumors, B - in lymph node metastasis and C - in primary tumors and/or lymph node metastasis

HR indicates hazard risk, CI95% - confidence interval 95%

Stromal ALDH1 expression evaluated in primary tumors appeared to be an independent prognostic marker of longer disease-free (Cox Regression model, HR = 0.851, CI95% 0.726–0.928, *p* = 0.047, *n* = 328) (Table 1A). When stromal ALDH1 was evaluated in lymph node metastasis, it occurred to be an independent predictor of longer disease-free (Cox Regression model, HR = 0.627, CI95% 0.454–0.865, *p* = 0.004, *n* = 79) and metastasis-free survival (Cox Regression model, HR = 0.405, CI95% 0.226–0.725, *p* = 0.002, *n* = 51) (Table 1B). When evaluated in primary tumors and/or lymph node metastasis, stromal ALDH1 was independent predictor of longer disease-free (Cox Regression model, HR = 0.806, CI95% 0.693–0.938, *p* = 0.005, *n* = 359) and metastasis-free survival (Cox Regression model, HR = 0.712, CI95% 0.555–0.913, *p* = 0.007, *n* = 204) (Table 1C).

### Associations of stromal ALDH1 expression with disseminated tumor cells

Immune and/or stromal cells are known to have an impact on tumor cell dissemination and metastases formation [33]. Therefore, ALDH1 expression was compared to the detection of DTCs in bone marrow and distant overt metastasis at the time of diagnosis or in the later time points of tumor disease.

**Figure 3:**
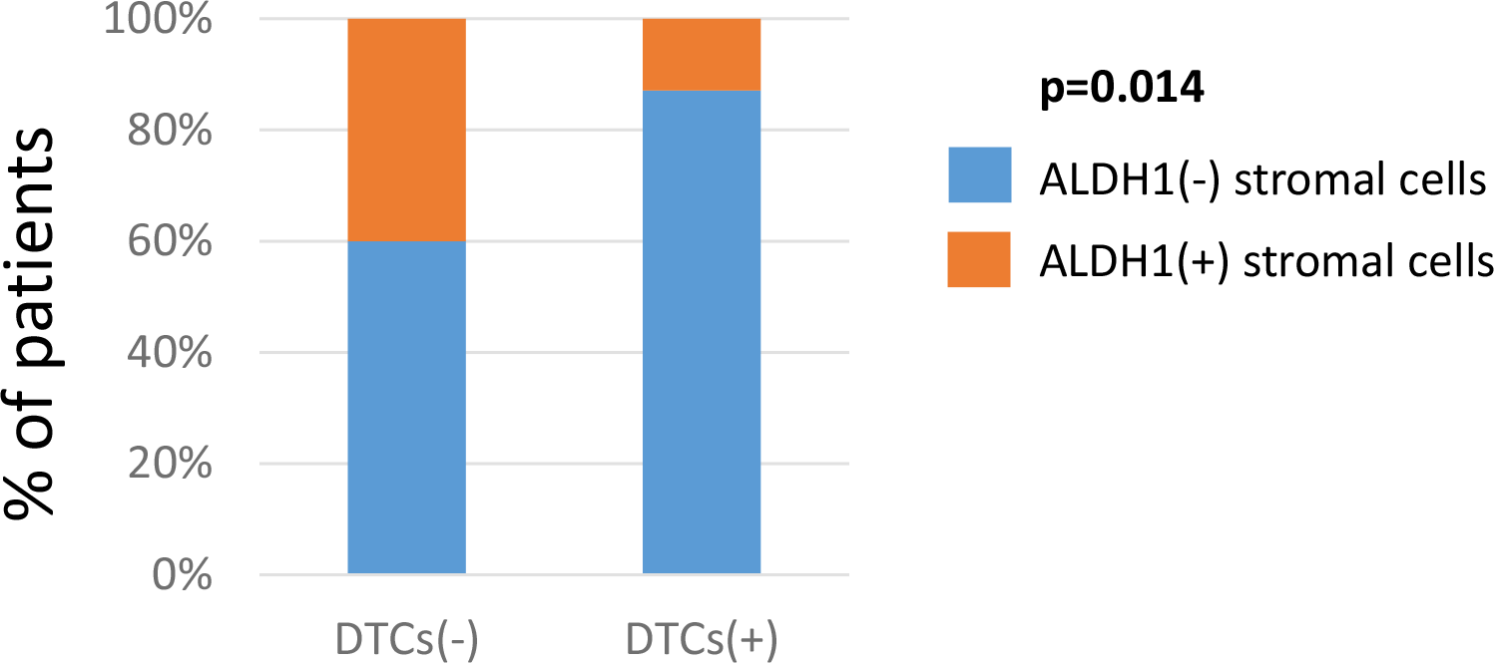
Comparison of stromal ALDH1 expression to tumor dissemination Correlation of stromal ALDH1 staining detected in lymph node metastasis to the presence of DTCs in bone marrow. DTC indicates disseminated tumor cell.

Stromal ALDH1 expression displayed in LNMs correlated inversely to the presence of DTCs in bone marrow of the analysed patients (Fisher exact test, *p* = 0.014) (Figure 3).

### ALDH1 expression in tumor cells

Seventy-three (19.5%) breast cancer patients were classified as positive for ALDH1 staining in tumor cells of primary tumors based on the mean 14.12 as cut-off. Twenty-two (21.6%) patients were positive for ALDH1 staining in tumor cells of LNMs. ALDH1 staining was localized in all cases in the cytoplasm of the analysed cancer cells (Figure 1). The frequency of tumor cells positive for ALDH1 ranged from 1 to 90% per tumor sample with a mean of 7% per primary tumor and 11% per LNM sample.

The expression of ALDH1 in tumor cells in LNMs matched to its expression in primary tumor (R^2^ = 0.410, *p* = 0.001): 45 (77.6%) cases had the same status of ALDH1 in tumor cells both in primary tumor and LNM. Of note, 7 (12.1%) patients had ALDH1-positive tumor cells only in LNM.

Expression of ALDH1 in tumor cells correlated to the molecular subtype of breast cancer. It appeared less frequently in luminal A tumors and more frequently in Her2-positive tumors (Chi^2^ = 9.701, *p* = 0.021) (Suppl. Table 1). Tumoral ALDH1 expression was also strongly associated with vascular invasion (Chi^2^ = 32.028, *p* < 0.001) (Suppl. Table 1) but did not correlate to any other clinico-pathological parameter. Tumoral ALDH1 expression evaluated in lymph node metastasis and primary tumors and/or lymph node metastasis indicated shorter metastasis-free (Kaplan-Meier log rank analysis, *p* = 0.008 and *p* = 0.012, respectively) in the stage I-III patients (Supl. Figure 1). It was independent predictor of shorter metastasis-free survival when evaluated in lymph node metastasis (Cox Regression model, HR = 24.426, CI95% 4.208–141.793, *p* < 0.001, *n* = 79) and primary tumor and/or lymph node metastasis (Cox Regression model, HR = 3.310, CI95% 1.264–8.663, *p* = 0.015, *n* = 204) (Table 1).

ALDH1 in tumor and stroma did not correlate with each other (Pearson correlation, R^2^ = 0.061, *p* = 0.201). Positive ALDH1 staining was observed exclusively in stromal cells of 41.6% patients. ALDH1 positivity in both tumor and stromal cells was found in 10.7%, whereas exclusively in tumor cells - in 7.5% patients.

### Associations of ALDH1-positive stromal cells to CD68 and HLA-DR expression

The composition of stromal cells in breast carcinomas is complex. Based on the literature (search criteria: cell morphology, involvement in tumor suppression and/or information about ALDH1 presence, if available), we limited our investigation to two types of immune cells (dendritic cells and tumor-associated macrophages) and their putative markers (CD68 and HLA-DR). CD68 staining marks the various cell types of the macrophage lineage [34], whereas HLA-DR expression is specific for immune cells presenting antigen (e.g. dendritic cells, macrophages) [35]. When co-expressed, CD68 and HLA-DR indicate macrophages M1 known to suppress tumor development [36].

**Figure 4:**
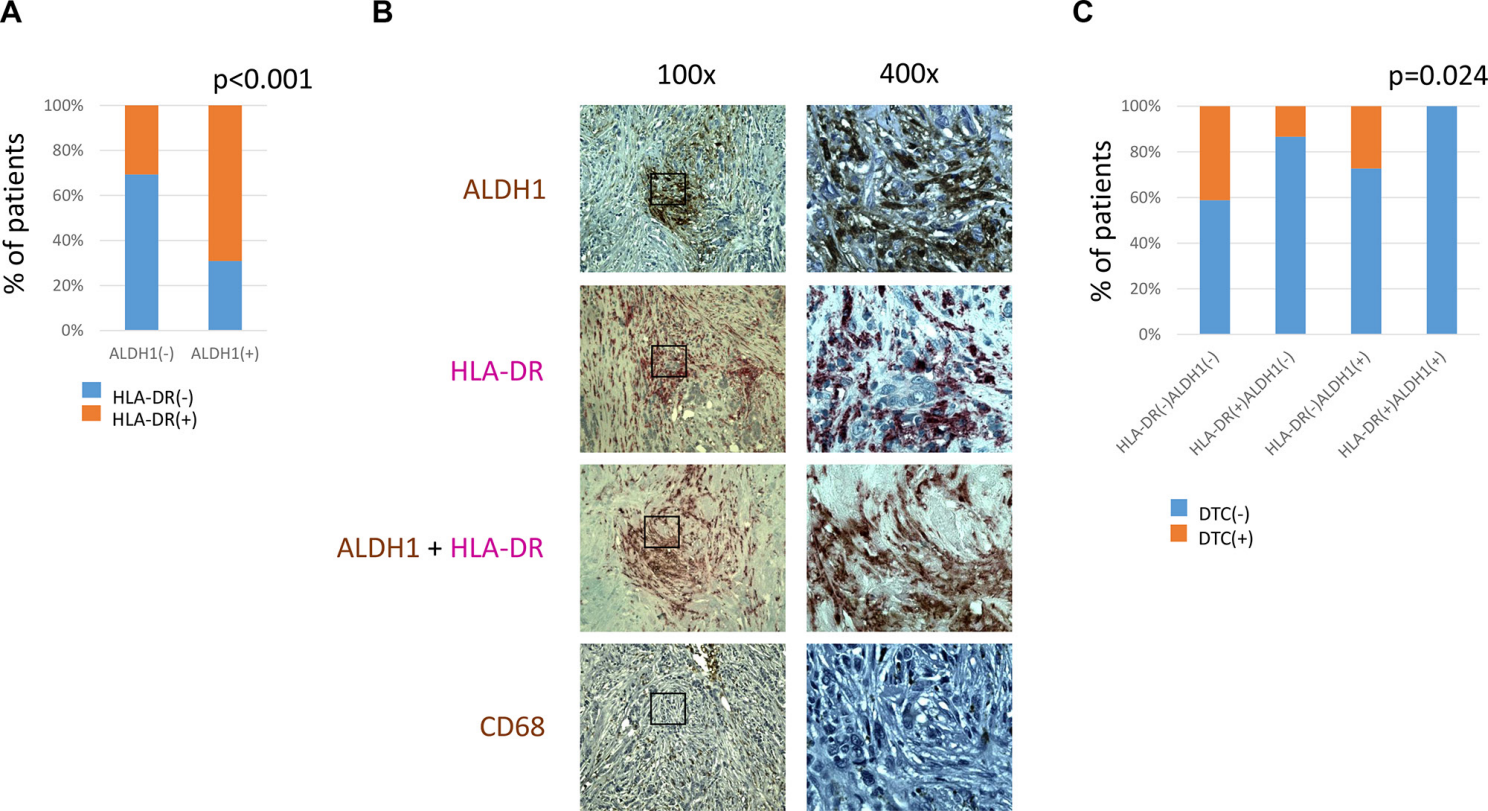
Comparison of ALDH1, CD68 and HLA-DR expression in stromal cells **A.** – correlation of ALDH1 and HLA-DR expression in stromal cells **B.** – representative pictures of parallel sections showing ALDH1, HLA-DR, ALDH1/HLA-DR and CD68 expression in the same region of breast cancer containing non-macrophage-like stromal cells, magnification 100x and 400x **C.** – correlation of HLA-DR/ALDH1 score and presence of DTCs in bone marrow. DTC indicates disseminated tumor cell.

One-hundred-seventeen tumor samples were simultaneously informative for CD68, HLADR and ALDH1 stainings. ALDH1 staining in stromal cells correlated strongly to HLA-DR-stromal positivity (Chi^2^ = 17.243, *p* < 0.001) (Figure 4A). Comparing the same regions of tumors on the serial sections, ALDH1 and HLA-DR co-localized in stromal cells of non-macrophage-like morphology (Figure 4B).

CD68 staining analysed alone correlated to clinical characteristics for more aggressive tumors (e.g., higher T and N status) and an unfavourable prognosis, whereas HLA-DR staining in stromal cells had no impact on patients’ outcome or tumor characteristics (data not shown).

HLA-DR/ALDH1 and CD68/ALDH1 combined scores were counted and compared to different clinico-pathological parameters and patients’ survival (*n* = 59). HLA-DR/ALDH1 score correlated to bone marrow status: patients who had stroma positive both for ALDH1 and HLA-DR staining had no DTCs (Chi^2^ = 9.422, *p* = 0.024) (Figure 4C). No other associations were found in this subanalysis.

### Associations of ALDH1 expression in stromal cells to the presence of retinoic acid and selected proteins determined in tumor cells

**Figure 5:**
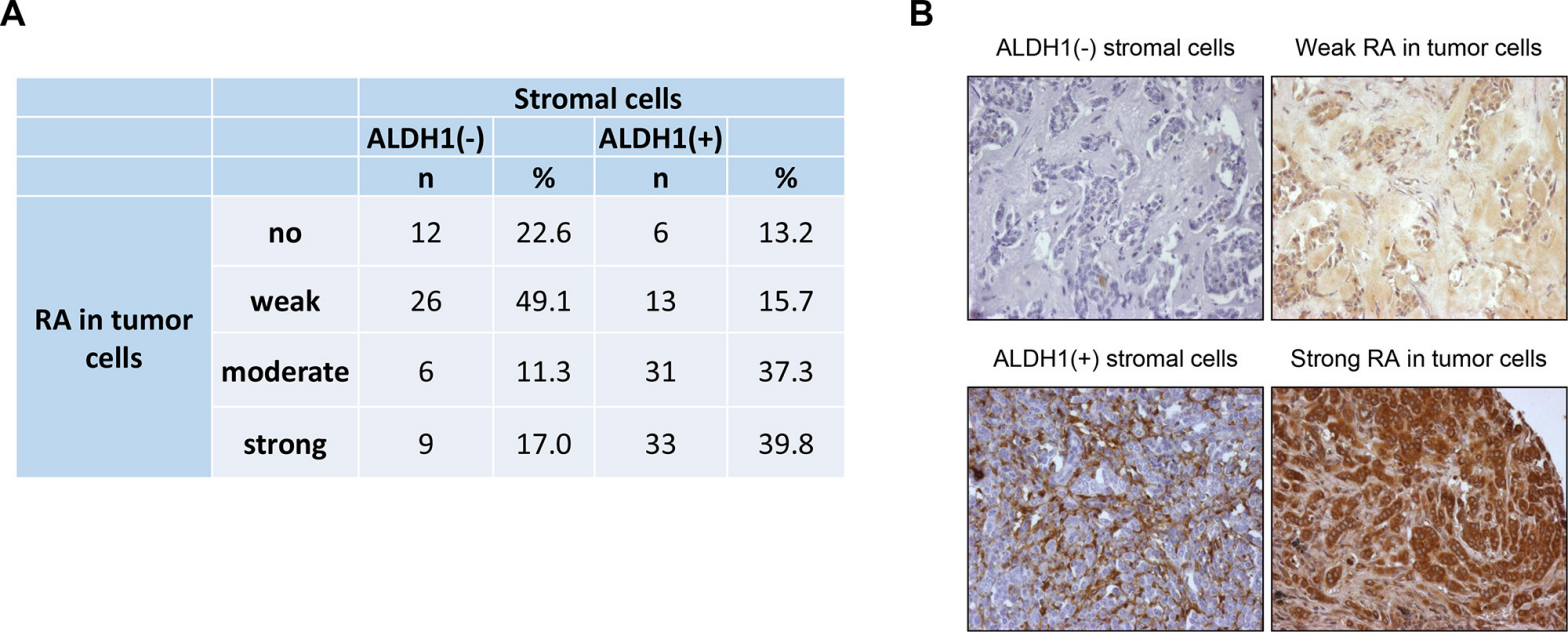
Comparison of stromal ALDH1 staining to presence of retinoic acid in tumor cells **A.** – correlation of stromal ALDH1 staining to presence of retinoic acid in tumor cells **B.** – representative pictures of parallel sections showing ALDH1 and retinoic acid staining in stromal and tumor cells, respectively.

One possible explanation for a differential clinical relevance of ALDH1 expression in tumor and stromal cells might be cell-specific involvement of ALDH1 protein in the synthesis of retinoic acid, an inducer of tumor cell differentiation and suppressor of tumor cell migration and proliferation [28–30]. To test whether tumors with ALDH1-positive stromal cells are characterized by different levels of retinoic acid, a subset of 136 primary breast cancer samples were analysed for the presence of this molecule in tumor and stromal cells. ALDH1-positive stromal cells were strongly associated with a higher intensity of retinoic acid staining in tumor cells (Chi^2^ = 31.973, *p* < 0.001) (Figure 5A, 5B).

Detailed comparison of ALDH1 stromal expression to selected molecular markers determined in tumor cells was performed in patients included in the Polish cohort (informative *n* = 140). The set included proteins indicating epithelial-mesenchymal transition (EMT) and/or (de-)differentiation of analysed tumors, both features which might be potentially regulated by retinoic acid. Deficiency of stromal ALDH1 was associated with increased expression of vimentin (Chi^2^ = 8.569, *p* = 0.036) (Table 2). It showed also borderline correlation to EMT phenotype and proliferation marker Ki-67 (Table 2).

**Table 2:** Detailed comparison of stromal ALDH1 expression to the presence of selected molecular markers in tumor cells

		ALDH1 (+) in stromal cells							
		no expression		in <10% of cells		in 10–50% of cells		in >50% of cells	
		*n*	%	*n*	%	*n*	%	*n*	%
**Ki-67**	**neg**	24	60.0	24	68.6	9	37.5	20	69.0
	**pos**	16	40.0	11	31.4	15	62.5	9	31.0
	**total**	128							
	***p*-value**	0.068							
**CK5/6**	**neg**	32	84.2	34	91.9	23	100.0	25	92.6
	**pos**	6	15.8	3	8.1	0	0.0	2	7.4
	**total**	125							
	***p*-value**	0.202							
**E-cadherin**	**neg**	12	33.3	9	26.5	6	25.0	10	40.0
	**pos**	24	66.7	25	73.5	18	75.0	15	60.0
	**total**	125							
	***p*-value**	0.623							
**Vimentin**	**neg**	29	76.3	35	89.7	26	100.0	26	89.7
	**pos**	9	23.7	4	10.3	0	0.0	3	10.3
	**total**	125							
	***p*-value**	**0.036**							
**EMT**	**no EMT: E-cad(+)Vim(−)**	19	55.9	22	64.7	18	75.0	14	52.0
	**partial EMT: E-cad(−)Vim(−) or E-cad(+)Vim(+)**	10	29.4	12	35.3	6	25.0	11	44.0
	**complete EMT: E-cad(−)Vim(+)**	5	14.7	0	0.0	0	0.0	1	4.0
	**total**	117							
	***p*-value**	0.092							

EMT indicates epithelial-mesenchymal transition

## DISCUSSION

So far ALDH1 expression has been described in tumor cells and associated with cancer cell stemness and worse clinical outcome. In the current study, the opposite effect was observed for ALDH1 expression in stromal cells, suggesting that ALDH1 might play a dual role in breast cancer progression. To the best of our knowledge, this is the first report on human material showing that stromal cells might be involved in regulation of tumor cell dissemination.

Tumoral ALDH1 expression was observed in 20% of breast cancer patients, which is in agreement with the literature data ranging from 18% to 56% [3, 4, 10, 18, 19, 24]. Correlations between ALDH1 protein expression in tumor cells and parameters of tumor aggressiveness were confirmed in the present study (i.e. vascular invasion, shorter metastasis-free survival), however, they were not so strongly manifested as in other studies.

The stromal ALDH1 expression was found in 53% of breast cancer patients. It appeared to be inversely correlated to tumor progression in the stage I-III patients. The current study is the first one showing that stromal ALDH1 staining is an independent favourable prognostic marker in an unselected cohort of breast cancer patients. This finding supports the concept that the tumor microenvironment plays a pivotal role in tumor progression.

Patients with tumors and/or lymph node metastasis positive for ALDH1 staining in stroma developed distant metastases later than patients with tumors without ALDH1(+) stroma. ALDH1-positive stromal cells of LNM correlated inversely with the presence of DTCs in bone marrow, a known adverse prognostic factor in breast cancer [37]. These observations support the idea that ALDH1-positive stromal cells in both primary tumor and lymph nodes might suppress the ability of tumor cells to further disseminate into distant organs. This finding is particularly interesting in the context of the current debate on the role of lymph nodes as potential incubators for disseminating tumor cells [38].

The nature of ALDH1-positive stromal cells has not been determined yet. It might be hypothesized that such cells are subpopulation of fibroblasts or immune cells recruited by the tumor. In the literature, however, non-tumor cells infiltrating tumors are mostly reported to reinforce their aggressiveness [33] and little is known about stromal cells attenuating outgrowth of a tumor. In the present study, ALDH1 stromal expression colocalized and was associated with HLA-DR staining detected in stromal cells of parallel TMA sections. Of note, HLA-DR staining alone did not provide any prognostic information, whereas combined HLA-DR/ALDH1 score correlated inversely to the presence of DTCs in bone marrow. This observation suggests that ALDH1-positive stromal cells might be dendritic cells known to attenuate tumor outgrowth. Future multimarker-based identification of stromal cells should define the precise subset of ALDH1-positive stromal cells suppressing tumor progression.

ALDH1 in tumor cells is believed to induce stem-cell properties and chemoresistance programs [1], whereas putative protective mechanism of ALDH1 in stromal cells is still unexplored. The antibody used in the current study detected potentially all isoforms of ALDH1 protein, while different isoforms might be differentially expressed in different compartments of a tumor. ALDH1 staining in stromal cells might hypothetically also result from the presentation of ALDH1 protein by dendritic cells.

Alternatively, it might be reasoned that ALDH1 expressed in different types of cells is involved in different molecular pathways. It can be speculated that ALDH1(+) stromal cells might synthesize and secrete retinoic acid into the tumor microenvironment [39, 40]. In turn, stromal cell-derived retinoic acid might induce tumor cell differentiation, inhibit proliferation and migratory abilities of tumor cells directly or via regulation of other immune cells [28–30, 32], which, in consequence, decreases the aggressiveness of a tumor and reduces its progression. In agreement with this model, in the present study, levels of retinoic acid were observed to be increased in tumor cells surrounded by ALDH1(+) stromal cells. Tumors with lower content of ALDH1-positive stromal cells were also characterized more frequently by vimentin expression, indicating a mesenchymal phenotype and increased migratory abilities of tumor cells. Future experimental studies are required to identify precise mechanisms behind this putative crosstalk between tumor and stroma cells in breast cancer.

## CONCLUSIONS

**Figure 6:**
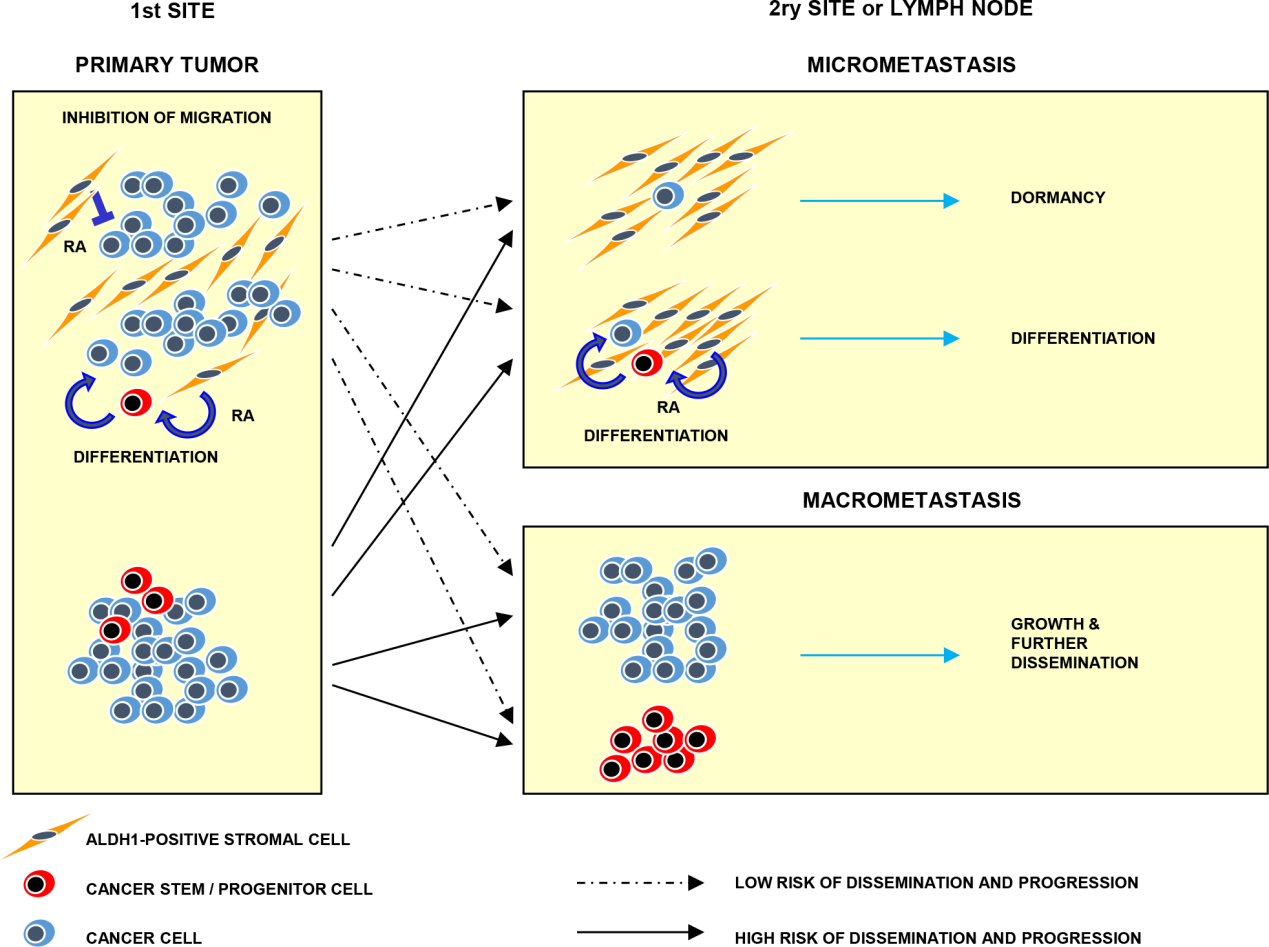
Model of hypothetical involvement of ALDH1(+) stromal cells in tumor progression ALDH1(+) stromal cells present at tumor primary sites might induce cancer (stem) cells differentiation and inhibit their migration via secretion of retinoic acid. At secondary sites they might suppress outgrowth of micrometastasis and secondary dissemination. Absence of ALDH1(+) stromal cells might lead to tumor progression and dissemination. RA indicates retinoic acid

The present findings suggest that ALDH1(+) stromal cells might act as local guardians of tumor cells reducing odds for tumor progression, possibly through the secretion of retinoic acid (Figure 6). Deficiency or decrease in the number of ALDH1(+) stromal cells might support tumor dissemination and development of overt metastases. Anti-aldehyde dehydrogenase therapeutic strategies are planned in different preclinical settings [41, 42], whereas our present study suggests that such drugs might exert adverse effects if the tumor contains ALDH1(+) stromal cells.

## MATERIALS AND METHODS

### Patients

Five-hundred-eighty-nine breast cancer patients were included in this study based on their signed informed consent form. The patients were treated by mastectomy or breast conserving surgery at the Medical University of Gdańsk and the Regional Cancer Center in Bydgoszcz during 2001–2008 (Poland, *n* = 178, named further as Polish Cohort), and University Medical Center Hamburg-Eppendorf during 1999–2006 (Germany, *n* = 411, named further as Hamburg Cohort). The patients received endocrine treatment, radiotherapy and/or chemotherapy according to the national guidelines. The variable clinico-pathological and molecular parameters were documented [43–45] (Suppl. Table 2–4). TNM staging was applied according to the American Joint Committee on Cancer 7^th^ Edition Recommendations, whereas molecular subtype classification was assigned based on immunohistochemical evaluation of estrogen receptor (ER), progesterone receptor (PgR) and Her2 as described [46]. Last follow-up for Polish Cohort was completed in August 2011 and for Hamburg Cohort - in August 2014. Of note, for survival analysis of the obtained results only stage I-III breast cancer patients were selected.

In addition to general clinico-pathological parameters, the presence of disseminated tumor cells (DTCs), later occurrence of overt metastases, metastasis-free survival and different therapy regiments were documented for patients included in the Hamburg Cohort (Suppl. Table 3). Selected patients from this cohort were examined also for further identification of stromal cells. DTCs in bone marrow were isolated and detected as described [47].

Patients included in the Polish Cohort were examined for different molecular markers determined in tumor cells (proliferation marker Ki-67, basal cytokeratin CK5/6, epithelial cell marker E-cadherin, basal and mesenchymal cell marker vimentin, as well as EMT defined as a switch between E-cadherin and vimentin) (Suppl. Table 4). In a very small subgroup of these patients (*n* = 61) information about presence of tumor cell microemboli in intratumoral vessels of tumors was also available (Suppl. Table 4).

**Figure 7:**
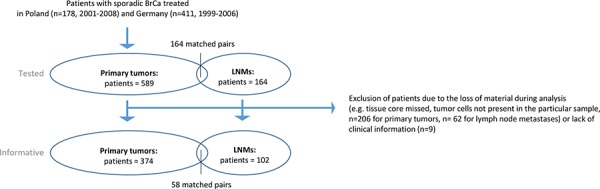
Study setting

The study was conducted according to REMARK study recommendations [48] (Figure 7) and in accordance with the Helsinki Declaration of 1975.

### TMA

Fourteen tissue microarrays (TMAs) with primary breast cancer were prepared as described before [43, 44]. Briefly, each TMA comprised of 0.6- (Hamburg Cohort, TMA *n* = 3) or 1.5-mm (Polish Cohort, TMA *n* = 11) diameter tissue cores obtained from formalin-fixed paraffin embedded breast cancer specimens. Cores of normal breast, colon, pancreas or tonsil tissues were introduced to TMAs as internal controls. Patients were represented by one (*n* = 355) or two (*n* = 243) fragment(s) of tumor (TMA tissue cores). One-hundred-ninety-nine fragments of breast cancer lymph node metastases (LNM) from 164 selected patients (1 to 4 samples per patient) were embedded into primary tumor TMAs (Hamburg Cohort) or constructed as 3 additional TMAs (Polish Cohort). TMA sections 4–6 μm thick were placed on charged polylysine-coated slides (Superfrost Plus, BDH, Germany) for further examination.

### Immunohistochemical detection of ALDH1, CD68, HLA-DR, retinoic acid and selected proteins

Deparaffinized TMA sections were treated for 5 min. in citrate buffer (pH 6.0, Biogenex, USA) at 120°C in a steamer. For ALDH1 and HLA-DR detection, specimens were incubated overnight at 4°C with mouse monoclonal anti-ALDH1 antibody (44/ALDH1, BD Biosciences, US) or rabbit monoclonal anti-HLA-DR antibody (EPR3692, Abcam) diluted 1:500 and 1:800, respectively, in Dako REAL™ Antibody Diluent (Dako, Denmark). For CD68 detection, specimens were incubated for 45 min. at RT with 1:100 dilution of mouse monoclonal anti-CD68 antibody (clone PG-M1, DakoCytomation, Denmark). Anti-retinoic acid monoclonal rabbit antibody (Abnova, US) was applied according to manufacturer’s protocol with minor modifications. Briefly, specimens were incubated with the antibody diluted 1:1000 in Dako REAL™ Antibody Diluent (Dako, Denmark) for 1.5 h at RT and overnight at 4°C after 30 min. of permeabilzation in 1xPBS/0.1 Tween.

All stainings were envisioned by DAKO ChemMate Detection Kit Peroxidase/DAB, Rabbit/Mouse (Dako, Denmark) and counterstained with hematoxylin (Merck, Germany). Double staining for ALDH1 and HLA-DR is described in Supplementary Data. CK5/6, E-cadherin, vimentin and Ki-67 expression was examined as described [40].

### Evaluation of the immunohistochemical stainings

Intensity (no, weak, moderate or strong) and subcellular localization of staining, as well as percentage of positive cells were documented for ALDH1 (in tumor cells), CD68 and HLA-DR (in stromal cells), and retinoic acid (in tumor and stromal cells). For semi-quantitative approach intensity of the staining was multiplied by percentage of the stained cells to result in index score of 0 to 300. ALDH1 expression in stromal cells was determined as no expression, moderate or strong expression in less than 10%, in 10–50% and more than 50% of stromal cells.

Two tumor samples (TMA tissue cores) from each patient were assessed individually. To evaluate overall score corresponding to one patient, maximal intensity or index score of ALDH1 staining in tumor cells and its minimal intensity in stromal cells was chosen from two analyzed tumor samples. If one tissue core was uninformative, the overall score corresponded to the remaining one. For the assessment of LNM the maximal index score in tumor cells and minimal staining intensity in stromal cells was chosen from all available LNM samples for one patient. Intratumoral heterogeneity was defined as difference in staining for tumoral or stromal ALDH1 between two tumor fragments of one patient.

Different biological, clinical and mathematical cut-offs of staining were tested for statistical analysis of the obtained results in comparison to clinico-pathological parameters and molecular data (Suppl. Table 5). Optimal cut-off for different stainings was selected as follows. For ALDH1 tumoral staining, the mean value of all index scores was used as a cut-off to determine negative (index score lower than mean) or positive expression (index score equal or greater than mean). For ALDH1 stromal staining results were classified as negative or positive according to the cut-off equal 10% positive cells. CD68 or HLA-DR positivity in stromal cells was established as for ALDH1 based on the cut-off equal mean value of all index scores in informative samples. CK5/6, E-cadherin, vimentin and Ki-67 expression in tumor cells was evaluated as described [49]. Epithelial-mesenchymal transition (EMT) in tumor cells was defined as a switch between E-cadherin and vimentin as: E-cad(+)vim(−) (no EMT), E-cad(−)vim(−) or E-cad(+)vim(+) (partial EMT) and E-cad(−)vim(+) (complete EMT).

### Statistics

Statistical analysis was performed with the usage of SPSS software version 22.0 licensed for University Medical Centre Hamburg-Eppendorf. Chi-square, and Fisher’s exact tests, as well as Pearson two-tailed correlation test and independent samples *t*-test were used as appropriate in order to compare the results to molecular factors or clinico-pathological parameters. Associations between protein expression profiles and disease-free, metastasis-free and overall survival were evaluated using Log Rank (Mantel Cox) test and Kaplan-Meier plot. To estimate hazard risk, Cox-Hazard-Potential regression analysis (CI 95%) was done. All results were considered statistically significant if *p* < 0.05 and highly statistically significant if *p* < 0.001.

## SUPPLEMENTARY INFORMATION FIGURE AND TABLES



## Abbreviations

ALDH1: aldehyde dehydrogenase 1
BM: bone marrow
DFS: disease free survival
DTC: disseminated tumor cell
LNM: lymph node metastasis
OS: overall survival
TMA: tissue microarray
