# Supplementary material for: Stromal expression of ALDH1 in human breast carcinomas indicates reduced tumor progression
Source: Oncotarget. 2015 Jul 20;6(29):26789–803. doi: 10.18632/oncotarget.4628 (PMC4694953; doi:10.18632/oncotarget.4628)
Supplement: Supplementary file 1 [file oncotarget-06-26789-s001.pdf]

SUPPLEMENTARY INFORMATION

IMMUNOHISTOCHEMICAL DETECTION OF ALDH1/HLA-DR DOUBLE STAINING

Deparaffinized TMA sections were treated for 5 min. in citrate buffer (pH 6.0, Biogenex, USA) at 120°C in steamer and incubated overnight at 4°C with rabbit monoclonal anti-HLA-DR antibody (EPR3692, Abcam) and mouse monoclonal anti-ALDH1 antibody (44/ALDH1, BD Biosciences, US) diluted 1:800 and 1:500, respectively, in Dako REALTM Antibody Diluent (Dako, Denmark). Primary antibodies were detected and envisioned with the usage of secondary anti-mouse antibody labelled with horseradish peroxidase diluted 1:100 followed by 10 min. incubation with DAB substrate diluted 1:50 (Dako, Denmark) and anti-rabbit antibody labelled with alkaline phosphatase diluted 1:40 followed by 20 min. incubation with new fuchsine. The specimens were counterstained with hematoxylin (Merck, Germany).

SUPPLEMENTARY FIGURE AND TABLES

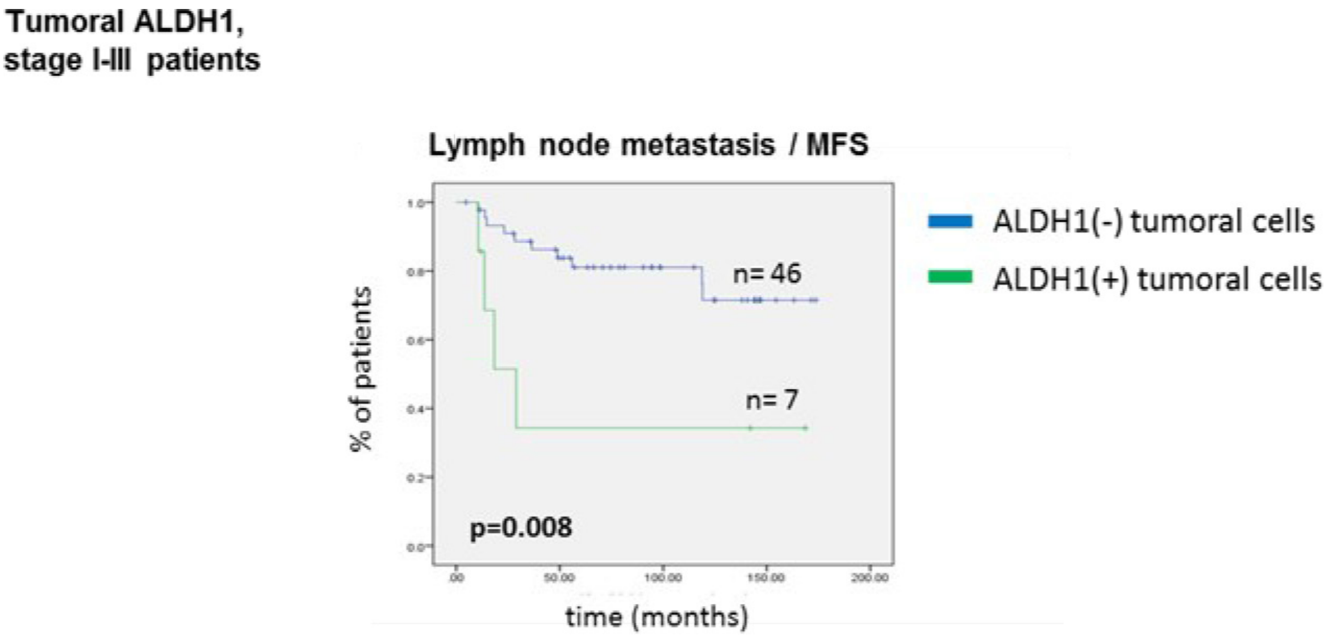

Supplementary Figure S1: Impact of ALDH1 expression in tumor cells of lymph node metastasis on survival of stage I-III breast cancer patients.

Supplementary Table S1. Comparison of stromal and tumoral ALDH1 expression to clinico-pathological data

Clinical, pathological and molecular parameters		ALDH1 stromal				ALDH1 tumoral			
		Neg		Pos		Neg		Pos	
		n	%	*n*	%	*n*	%	*n*	%
**Age**	**<median**	79	47.3	96	54.9	143	51.3	32	50.8
**(years)**	**> = median**	88	52.7	79	45.1	136	48.7	31	49.2
	***p*-value**	0.163				0.947			
**T status**	**T1**	73	43.7	84	48.3	133	47.7	24	38.7
	**T2**	76	45.5	75	43.1	119	42.7	32	51.6
	**T3**	8	4.8	9	5.2	14	5.0	3	4.8
	**T4**	10	6.0	6	3.4	13	4.7	3	4.8
	***p*-value**	0.638				0.613			
**N status**	**N0**	96	57.8	100	58.1	166	60.4	30	47.6
	**N1-3**	70	42.2	72	41.9	109	39.6	33	52.4
	***p*-value**	0.954				**0.065**			
**M status**	**M0**	198	97.5	218	99.5	340	98.3	76	100.0
	**M1**	5	2.5	1	0.5	6	1.7	0	0.0
	***p*-value**	0.110 (F)				0.597 (F)			
**TNM**	**IA**	52	31.7	65	38.0	98	35.9	19	30.6
**staging**	**IIA**	53	32.3	49	28.7	87	31.9	15	24.2
	**IIB**	27	16.5	31	18.1	43	15.8	15	24.2
	**IIIA**	14	8.5	16	9.4	23	8.4	7	11.3
	**IIIB**	10	6.1	5	2.9	12	4.4	3	4.8
	**IIIC**	3	1.8	4	2.3	4	1.5	3	4.8
	**IV**	5	3.0	1	0.6	6	2.2	0	0.0
	***p*-value**	0.386				0.234			
**Grade**	**G1**	17	10.3	30	17.5	36	13.2	11	17.5
	**G2**	92	55.8	82	48.0	143	52.4	31	49.2
	**G3**	56	33.9	59	34.5	94	34.4	21	33.3
	***p*-value**	0.126				0.675			
**Vascular**	**no**	86	71.7	92	78.0	167	81.1	11	34.4
**invasion**	**yes**	34	28.3	26	22.0	39	18.9	21	65.6
	***p*-value**	0.263				**<0.001**			
**Recurrence**	**no**	113	71.5	140	80.9	203	75.5	50	80.6

(*Continued*)

Clinical, pathological and molecular parameters		ALDH1 stromal				ALDH1 tumoral			
		Neg		Pos		Neg		Pos	
		n	%	*n*	%	*n*	%	*n*	%
	yes	45	28.5	33	19.1	66	24.5	12	19.4
	p-value	**0.044**				0.386			
**Death**	no	132	81.0	156	89.1	231	84.0	57	90.5
	yes	31	19.0	19	10.9	44	16.0	6	9.5
	p-value	**0.035**				0.239 (F)			
**Her2**	neg	141	92.8	150	95.5	241	94.9	50	90.9
	pos	11	7.2	7	4.5	13	5.1	9	9.1
	p-value	0.297				0.254			
**HR**	neg	38	22.9	35	20.2	59	21.2	14	23.0
	pos	128	77.1	138	79.8	219	78.8	47	77.0
	p-value	0.551				0.766			
**Molecular**	Luminal A	42	31.6	44	31.2	77	33.8	9	19.6
**subtype**	Luminal B	56	42.1	63	44.7	96	42.1	23	50.0
	Her2-pos	3	2.3	2	1.4	2	0.9	3	6.5
	Basal-like	32	24.1	32	22.7	53	23.2	11	23.9
	p-value	0.935				**0.021**			

F indicates Fisher exact test, otherwise Chi square test.

Supplementary Table S2. Clinico-pathological characteristics of breast cancer patients included in the study

Clinical, pathological and molecular parameters	Status	*n*	%
**Age (years)**	median=58	58	
	range	27-86	
	<median	284	49.6
	>=median	289	50.4
	total	573	
**T status**	T1	276	48.3
	T2	241	42.2
	T3	30	5.3
	T4	24	4.2
	total	571	
**N status**	N0	332	58.3
	N1-3	237	41.7
	total	569	
**M status**	M0	566	97.6
	M1	14	2.4
	total	580	
**TNM staging**	IA	200	35.8
	IIA	171	30.6
	IIB	92	16.5
	IIIA	47	8.4
	IIIB	21	3.8
	IIIC	13	2.3
	IV	14	2.5
	total	558	
**Grade**	well differentiated (G1)	78	13.9
	moderately differentiated (G2)	298	52.9
	poorly differentiated (G3)	187	33.2
	total	563	
**Vascular invasion**	No	360	82.6
	Yes	76	17.4
	total	436	
**Recurrence**	No	466	82.5
	Yes	99	17.5
	total	565	

(*Continued*)

Clinical, pathological and molecular parameters	Status	*n*	%
**Death**	No	496	88
	Yes	68	12
	total	564	
**Her2**	negative	483	94.3
	positive	29	5.7
	total	512	
**HR**	negative	117	20.5
	positive	454	79.5
	total	571	
**Molecular subtype**	Luminal A	158	34.6
	Luminal B	194	42.5
	Her2-postive	6	1.3
	Basal-like	98	21.5
	total	456	

Note that due to the missing values not all numbers sum up to 589 cases.
HR indicates hormone receptor

Supplementary Table S3. Clinico-pathological and treatment characteristics of breast cancer patients included in Hamburg Cohort

	total	*n*	%
**DTC status**	**neg**	297	74.3
**in bone marrow**	**pos**	103	25.8
	**total**	400	
**Metastatic relapse**	**no**	320	85.6
	**yes**	54	14.4
	**total**	374	
**Chemotherapy**	**no**	150	37.8
	**yes**	247	62.2
	**total**	397	
**Radiotherapy**	**no**	81	20.3
	**yes**	319	79.8
	**total**	400	
**Endocrine therapy**	**no**	99	24.9
	**yes**	298	75.1
	**total**	397	

Note that due to the missing values not all numbers sum up to 411 cases.

Supplementary Table S4. Molecular and clinico-pathological characteristics of breast cancer patients included in Polish Cohort

	total	*n*	%
**Ki-67**	**neg**	96	58.9
	**pos**	67	41.1
	**total**	163	
	**missing**		
**CK5/6**	**neg**	139	91.4
	**pos**	13	8.6
	**total**	152	
	**missing**		
**E-cadherin**	**neg**	45	30.8
	**pos**	101	69.2
	**total**	146	
	**missing**		
**Vimentin**	**neg**	143	89.4
	**pos**	17	10.6
	**total**	160	
	**missing**		
**EMT**	**E-cad(+)Vim(−)**	90	62.5
	**E-cad(−)Vim(−)**	38	26.4
	**E-cad(+)Vim(+)**	10	6.9
	**E-cad(−)Vim(+)**	6	4.2
	**total**	144	
	**missing**		

Note that due to the missing values not all numbers sum up to 178 cases.
EMT indicates epithelial-mesenchymal transition, E-cad – E-cadherin, vim - vimentin

Supplementary Table S5. Different cut-offs tested in the current study

**Cut-off for ALDH1 in tumor cells based on**	**neg vs. pos**
intensity	no vs. weak vs. moderate vs. strong
intensity	no, weak vs. moderate, strong
intensity	no, weak, moderate vs. strong
intensity	no vs. weak, moderate, strong
% of positive cells	≥1%
% of positive cells	≥5%
**index score (maximal result for two tumor samples)**	**<mean index score vs. > mean index score**
index score (maximal result for two tumor samples)	<median index score vs. > median index score
index score (sum for two tumor samples)	<mean index score vs. > mean index score
index score (sum for two tumor samples)	<median index score vs. > median index score
**Cut-off for ALDH1 in stromal cells based on**	**neg vs. pos**
index score	no expression vs. expression in <10% of stromal cells vs. expression in 10-50% of stromal cells vs. expression in >50% of stromal cells
index score	no expression vs. expression in >1% of stromal cells
**index score**	**expression in <10% of stromal cells vs. expression in >10% of stromal cells**
index score	expression in <50% of stromal cells vs. expression in >50% of stromal cells
